# Married women's decision making power on modern contraceptive use in urban and rural southern Ethiopia

**DOI:** 10.1186/1471-2458-11-342

**Published:** 2011-05-19

**Authors:** Binyam Bogale, Mekitie Wondafrash, Tizta Tilahun, Eshetu Girma

**Affiliations:** 1 Department of Public Health, Arba Minch University, Arba Minch, Ethiopia; 2 Department of Population and Family Health, Jimma University, Jimma, Ethiopia; 3 Department of Health Education and Behavioral Sciences, Jimma University, Jimma, Ethiopia

## Abstract

**Background:**

Women in developing countries are either under collective decision making with their partners or completely rely on the male partner's decision on issues that affect their reproductive live. Identifying the major barriers of married women's decision making power on contraceptive use has significant relevance for planning contextually appropriate family planning interventions. The objective of this study was to determine current modern contraceptive practices and decision making power among married women in Tercha Town and surrounding rural areas of Dawro zone, Southern Ethiopia.

**Methods:**

Community based comparative cross-sectional design with both quantitative and Qualitative study has been employed in March and April 2010. The respondents were 699 married women of child bearing age from urban and rural parts of Dawro zone. After conducting census, we took the sample using simple random sampling technique.

**Results:**

Current modern contraceptive use among married women in the urban was 293 (87.5%) and 243 (72.8%) in rural. Married women who reside in urban area were more likely to decide on the use of modern contraceptive method than rural women. Having better knowledge about modern contraceptive methods, gender equitable attitude, better involvement in decisions related to children, socio-cultural and family relations were statistically significant factors for decision making power of women on the use of modern contraceptive methods in the urban setting. Better knowledge, fear of partner's opposition or negligence, involvement in decisions about child and economic affairs were statistically significant factors for better decision making power of women on the use of modern contraceptive methods in the rural part.

**Conclusions:**

High level of current modern contraceptive practice with reduced urban-rural difference was found as compared to regional and national figures. Urban women had better power to make decisions on modern contraceptive than rural women. Modern family planning interventions in the area should be promoted by considering empowering of women on modern contraceptive use decision making.

## Background

Africa's population, currently growing faster than any other major region, is projected to account for 21 percent of world population by 2050, up from just 9 percent in 1950 [1]. The 2007 Population and Housing Census results showed that the population of Ethiopia grew at an average annual rate of 2.6 percent and a total population of 73.9 million. Southern Nation Nationalities and People's Region, where the study area located, the annual growth rate is moderately higher than the national average at 2.9 percent [2].

According to Ethiopian Demographic and Health Survey (EDHS) 2005 report, the maternal mortality ratio for Ethiopia was 673 deaths per 100,000 live births [3]. Addressing family planning unmet need in Ethiopia is expected to avert 12,782 maternal deaths and more than 1.1 million child deaths by the target date of 2015 [4]. There is high fertility rate in Ethiopia (total fertility rate of 5.4) as compared to other developing countries [3,5].

In the context of family planning, the concept of women's empowerment is generally associated with a variety of elements that range from delayed marriage, smaller families, access to accurate information, the ability to discuss freely about their family planning needs with spouses and other members of the household and the community. Being able to make independent decisions on fertility regulation including going out of living boundaries to seek contraceptive supplies is also among these elements [6].

Family planning method use can help ensure healthiest timing and spacing of pregnancy, hence, regulating fertility. As fertility falls, so do infant, child, and maternal mortality. Women spend decreasing proportions of their lifetimes giving birth and caring for young children [7]. Contraception plays a key role in decreasing maternal mortality. They provide significant protection for women by preventing unintended pregnancies, which often end in unsafe abortions [8].

Despite the high level of knowledge (88% of currently married women and 93% of men knowing at least one method of contraception), the contraceptive prevalence rate (CPR) for married Ethiopian women is 15% (southern nations = 11.4%), which is far below the nation goal of the Ethiopian Population Policy to be attained by the year 2015(44.0%) [9]. In addition to these, the contraceptive prevalence is more than four times higher in urban than in rural areas [7].

Decisions about contraceptive use and childbearing may be confounded by unequal power relations, especially in more patriarchal societies [10]. Study in Ethiopia showed that because of the male dominance in the culture, women would be forced to bear large number of children [11]. Studies in sub-Saharan Africa showed also that secret use of contraceptives among women accounts for between 6 and 20% of all contraceptive use which indicates problem of decision making power of women on contraceptive use [12,13].

It is expected that women contraceptive decision making power to be less in a rural community where women's literacy status is very low and economic dependence is high [Bezabih T: Assessment of the determinants of modern contraceptive use in a Dawro community, Ethiopia: submitted]. In contrast to this finding, studies in Ethiopia found that rural women were more likely than urban women to make an independent decision on current use of contraceptive [3]. Therefore, the objective of this study was to measure married women's decision making power on the use of modern contraceptive method and reveal the differences on decision making power among urban and rural married women in Dawro Zone, Ethiopia.

## Methods

### Study area and setting

Community based comparative cross-sectional study was conducted on March and April 2010. The study was done in Dawro Zone which is found in the southern part of Ethiopia. The capital of the zone is Tercha town which is located 505 km south-West of Addis Ababa, the capital of Ethiopia. The total population of the area was 492,742. From the total population, 242,000 were female and 114,808 were in the reproductive age group [2]. Majority of the ethnic group is Dawro.

### Sampling

A sample of 672 Urban (336) and rural (336) married women of reproductive age group was determined using Epi Info version 3.3.2. It was determined by taking Level of significance (0.05), Power (0.80), Proportion of independent decision making on modern contraceptive use by urban married women (0.19) and rural women (0.29). For non-response 10% of the sample was added. Before the actual data collection, the questionnaire was pre-tested on 5% (34 women) of similar population in the nearby district. Census was conducted in two urban and five rural kebeles (the lowest administrative unit) which were found around 10 kilometers radius with Tercha zonal Hospital. The census was carried out to identify households with married women of reproductive age group in the kebeles in order to generate a sampling frame. After proportional allocation to the number of eligible women in each kebele, study units were identified with simple random sampling technique. Eight sessions of focus group discussions were undertaken among purposively selected married men and women from both urban and rural settings. The sample size was determined based on information saturation. Each focus group discussion consisted of 6-12 members. All the focus group discussions were moderated by principal investigators with the assistance of one trained note taker. Each focus group discussions lasted approximately an hour.

### Method of data collection

the quantitative study was done with face to face interview using the pre-tested questionnaire. The quantitative data was collected by diploma holder trained female nurses. We used female data collectors since most female in the study area might prefer to discus reproductive health matters openly with female than male. We have done qualitative study with focus group discussion method of data collection. We used focus group discussion to explore ideas of male partners and married women decision making power on contraceptive use and triangulate with the quantitative study. Hence, it has its own value on the quality and the validity of the finding. Each focus group discussion was tape recorded and note was taken during the discussion.

### Measurement

Both the quantitative and the qualitative instruments were prepared first in English then translated and administered in Dawrogna (the local language) for data collection. Language expert in Dawrogna has translated the instruments. To check whether the translation was consistent with the English meanings the instruments were back translated from Dawrogna to English by other language expert. The quantitative instrument contained items about socio demographic variables, utilization of modern contraceptive methods, decision making power on its use and factors affecting their ability to make decision. The quantitative study instrument was adopted from other literature [Bezabih T: Assessment of the determinants of modern contraceptive use in a Dawro community, Ethiopia: submitted]. Without including the socio-demographic parts, the overall internal reliability (coefficient alpha) of the instrument in this study was 0.82. In this study, modern contraceptive methods refer to methods of child spacing or birth control other than natural methods (abstinence, basal body temperature, cervical mucosa, and symptom-thermal and withdrawal methods).

To measure married women decision making power on modern contraception use, scores have been developed for three sets of women: current users, Ever users and None-users. **Current users**: six questions were asked to make mean score. After computing the total, score above mean is said to have better decision-making power. **Ever uses**: Those who used modern contraceptive at least once in their life time but currently not using; six questions were asked and the mean score above mean is said to have better decision making power. **Non-users**: if their main reason for non-use is opposition from others the value was assigned as 0 and 1 if otherwise. Finally, married women's decision-making in contraceptive use among study units was set as binary outcome variable by merging the three groups of women together those scored above the mean are those who are able to make decision on modern contraceptive use and who scored below the mean are unable to make decision on modern contraceptive.

To measure the degree of women's involvement in domestic decision-making, under three subheadings: decisions regarding children, Economic decisions and decisions related to Social, Cultural and Family Relations, which comprises 18 questions were used with 0-2 values. Score greater than or equal to five for decisions related to children and economic affairs and score greater than or equal to eight for socio-cultural and family relation decision making were considered as better involved in domestic decision making. Score below this value was categorized as weaker involvement in domestic decision making. Ten likert scale items were used to measure attitude on gender equity [Example: (A woman needs her husband's permission to use any contraceptive method) with a possible reposes of (agree, neutral or disagree)]. Score above 80% was considered as having gender equitable attitude otherwise not equitable attitude [Bezabih T: Assessment of the determinants of modern contraceptive use in a Dawro community, Ethiopia: submitted]. Nine knowledge questions were used to measure knowledge on modern family planning. Based on the summation score, individuals who scored above 70% were considered as having better knowledge on family planning otherwise not better knowledge [Bezabih T: Assessment of the determinants of modern contraceptive use in a Dawro community, Ethiopia: submitted]. We used focus group discussion guide for the qualitative study. The guide contains probing topics related with modern contraceptive decision making.

### Data analysis

The collected data was cleaned and fed to Statistical package for social sciences (SPSS) version 16.0. Descriptive analysis was carried out for each of the variables. Bivariate and multivariate analysis was done for the independent variables with the outcome variable. Variables which remain statistically significant in Bivariate analysis were entered to Multivariate Logistic regression model to get the final model.

Data from the focus group discussion was translated and transcribed to English and categorized accordingly to main thematic areas manually. We have read the data repeatedly. Finally the findings were triangulating with the quantitative findings.

### Ethical considerations

Ethical clearance was obtained from Jimma University; college of Public Health and Medical Sciences Ethical Committee. Informed consent was obtained from each study participant.

## Results

### Socio-demographic characteristics

The total response rate of the study was 669 (99.6%). The median age of the respondent was 26 (+/-5.6) years in urban and 27 (+/- 6.2) years in rural areas. Most of the respondents in both settings were in the age group 25-29. Among the rural participants 318 (95.2%) and 183 (54.6%) of urban women were house wife in their occupation. The majority subjects 186 (55.7%) in rural and only 17 (5.1%) urban study subjects were unable to read and write (Table 1)

**Table 1:** Socio-demographic variables of married women in reproductive age in Tercha Town and rural areas of Dawro zone, Southern Ethiopia, 2010 (N = 669)

Characteristics	Urban (%)	Rural (%)	Total (%)
**Age Group**			
**15-19**	31(9.3)	27(8.1)	58(8.7)
**20-24**	90(26.9)	73(21.9)	163(24.4)
**25-29**	123(36.7)	118(35.3)	241(36.0)
**30-34**	54(16.1)	50(15.0)	104(15.5)
**35-39**	28(8.4)	49(14.7)	77(11.5)
**40-44**	7(2.1)	15(4.5)	22(3.3)
**45-49**	2(0.6)	2(0.6)	4(0.6)
**Religion**			
**Orthodox**	110(32.8)	57(17.1)	167(25.0)
**Protestant**	218(65.1)	256(76.6)	474(70.9)
**Catholic**	4(1.2)	0 (0.0)	4(.6)
**Others (Muslim, Traditional)**	3(.9)	21(6.3)	24(3.6)
**Respondent's Educational status**			
**Cannot read and write**	17(5.1)	186(55.7)	203(30.3)
**1-6 grade completed**	77(22.9)	95(28.4)	172(25.7)
**7-10 grade completed**	119(35.5)	46(13.8)	165(24.7)
**10-12 grade completed**	21(6.3)	3(0.9)	24(3.6)
**Higher education completed**	47(14.0)	0 (0.0)	47(7.0)
**Occupation**			
**House wife**	183(54.6)	318(95.2)	501(74.9)
**Government employee**	107(31.9)	9(2.7)	116(17.3)
**Merchant**	33(9.9)	3(0.9)	36(5.4)
Others**	12(3.6)	4(1.2)	16(2.4)

**students, Daily Laborers, preachers

### Reproductive health characteristics

The mean age at first marriage in the urban was 18.92 (+/- 3.23) years where as in the rural was 17.07 (+/-2.48) years. From the total respondents, 614 (91.8%) have given birth at least once in their life time. Around 78 (11.7%) encountered at least one child death of which 69 (89%) were in rural community. In both groups male child is preferred to female 339 (50.7%) and significant number of the respondents 305 (45.6%) did not mind about sex preference.

### Knowledge, attitude and practice on modern contraceptives

Around 333 (99.4%) urban and 330 (98.8%) rural women have ever heard about modern contraceptive methods and knew at least one modern female contraceptive method. The major source of information in both settings was health professionals [310 (92.8%) and 213 (63.6%)] at rural and urban respectively. From specific modern family planning methods, frequently mentioned type was injectables 291 (86.9%) in urban and 319 (95.5%) in rural followed by pills 175 (52.1%) respondents in urban and 224 (67.2%) rural respondents. About 77.4% of urban and only 36.9% of rural women knew the presence of male modern contraceptive. Out of those who know the presence of male modern contraceptive types, 636 (95%) mentioned condom only at both settings. Generally, 155 (46.3%) of the urban and 123 (36.8%) of the rural women of reproductive age group were knowledgeable about modern contraceptive methods. A focus group participant said that "*If I have known about contraceptive before, I would have not born this all children.... My children would have been stronger and I could have been also stayed younger*" [A mother of five children from rural area]

**Figure 1:**
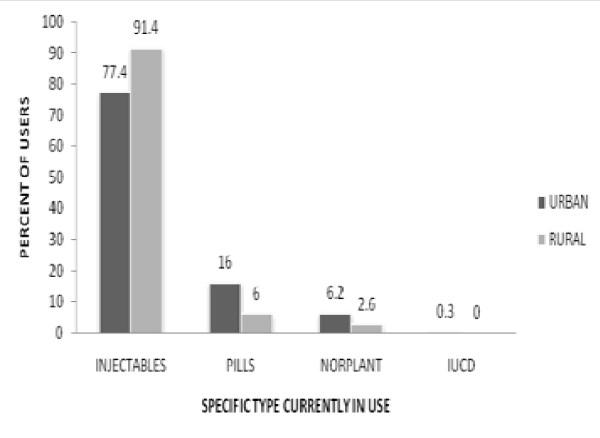
**Modern contraceptive method use in urban and rural areas of Dawro zone, SNNPR, 2010**.

Current modern contraceptive use among the study subjects was 293 (87.5%) among urban and 243 (72.8%) among rural women. Among the total population, 560 (83.7%) used injectables followed by 77 (11.5%) pills and 31 (4.6%) Norplant (Figure 1). Rural women were around three times more likely not to use modern contraceptive [OR- 2.7, 95% CI (1.78, 4.06)] compared with urban women. Some of the rural women aggressively opposed the use of permanent methods as it is an act against God. "*Health extension workers told us about cutting off the Uterus but I think this is making some one crippled....I want to die with my whole organ intact*!" [36 years old woman]. This idea was also supported by male rural participants. The majority of the respondents 327 (97.6%) in urban and 321 (96.1%) in rural reported that they used contraceptives without hiding their husbands. A mother of seven children and currently pregnant women said that every woman should use in compliance of her husband since if things get worse; "*he is the one who take responsibility". *The participants were asked what if the woman wants to use but her partner opposes? Majority of rural women agreed that they have to accept his decision. Some of them also replied as they will use without his knowledge. As 46 year old men said "*Now everybody accepted limiting family size since the economy worsens. However, the final say should lie on the hands of men. If the neighbors hear that the woman is using modern contraception without the permission of her husband, he will be seen as weak in the community which no man allows in his life...therefore, she must follow his decision, this is the tradition we lived and became old". *Whereas; the urban women pointed about accusation of violation of their personal right but cautious if the woman is economically dependent on her partner.

### Domestic decision making power

The overall decision-making power in urban areas tends to be joint 225 (67.06%) but in rural areas it is the husband without involving his wife 153 (45.83%). Among the respondents with joint decision makers, in conditions where their idea did not coincide or their decision were in conflict, the husband's decision override 331 (98.7%) in rural and 305 (91.2%) in urban. Small amount 43 (6.45%) of respondents replied they can independently decide in children related issues in both settings. Economic decisions taken by wife only showed relatively higher percentage in rural than urban; on the other hand decisions related to socio-cultural and family relations, the reverse holds true. Majority of the focus group participants agreed that mutual discussion is relevant but practically domestic decisions are male dominated especially in the rural part (Table 2).

**Table 2:** Domestic decision-making among married women of reproductive age group in Urban and rural areas of Dawro Zone, Southern Ethiopia, 2010.

		Who make decisions No (%)		
		
Settings	Decisions	Wife only	Jointly	Husband only
	Children related	22(6.4)	53(75.6)	60(18.0)
**Urban (N = 335)**	Economic	37(11.1)	242(72.2)	56(16.7)
	Socio-cultural & family relations	66(19.7)	179(53.4)	90 (26.9)
**Rural (N = 334)**	Children related	22(6.5)	204 (61.1)	108 (32.4)
	Economic	64(19.2)	107(32.1)	163(48.7)
	Socio-cultural & family relations	62(18.5)	84 (25.1)	188 (56.4)

### Contraception decision making

more than half of the respondents 360 (53.8%) reported that they can decide on the use of modern contraception. The proportion was higher in urban 214 (64%) than the rural 144 (43.1%). Urban women were also more likely to decide on modern contraceptive use than the rural women [OR- 2.4, 95% CI (1.75, 3.27)]

### Factors affecting modern contraceptive decision making power

in the urban area, women who were knowledgeable about the over all aspects of modern contraceptive methods were more likely to decide on its use [OR-3.6, 95% CI (2.13, 6.16)]. Women with gender equitable attitude in the urban were four times more likely to decide on the use of modern contraceptives as compared to those who showed inequitable attitude [OR- 4.0, 95% CI (2.36, 6.81)]. In urban areas women who has better involvement in decisions about children, socio-cultural and family relations were more likely to decide on the use of modern contraceptive not less than two fold with [OR-3.0, 95% CI (1.58,5.53)] and [OR-2.1, 95% CI (1.19,3.86)] respectively (Table 3).

**Table 3:** Factors contributing for modern contraceptive decision making power to urban residents, Dawro Zone, SNNPR, 2010

**Variables**	**Able to decide on modern contraceptive use**		**AOR (95.0% C.I)**
		
	**Yes**	**No**	
**Knowledge modern contraceptive**			
**Less knowledgeable**	96(53.5)	84(46.5)	1
**Knowledgeable**	115(74.4)	40(25.6)	3.6 (2.13,6.16)
**Gender Attitude**			
**Gender inequitable attitude**	100(55.9)	79(44.1)	1
**Equitable attitude**	116(74.4)	40(25.6)	4.0 (2.36,6.81)
**Decisions Related To Children**			
**Low involvement**	65 (50.0)	65 (50.0)	1
**Better involvement**	152 (74.1)	53 (25.9)	3.0 (1.58,5.53)
**Decisions related to socio-cultural and family relations**			
**Low involvement**	60(52.6)	54(47.4)	1
**Better involvement**	157(71.0)	64(29.0)	2.1 (1.19,3.86)

Whereas among rural respondent's, women who has better involvement in decisions about children [OR- 23.3, 95% CI (9.52, 56.91)] and those who were knowledgeable about the over all aspects of modern contraceptive methods [OR-6.8, 95% CI (3.28,13.91)] were more likely to decide on the use of modern contraceptive. But rural women who perceive that their partner's reaction on modern contraceptive use would be unsupportive [OR-5.4, 95% CI (1.20, 24.47)] and those who did not know their partner reaction [OR- 11.1, 95% CI (4.18, 29.57)] were more likely to decide on the use of modern contraceptive method than those who perceive their partner reaction would be supportive (Table 4). Modern family planning use decision making did not have significant statistical different with age groups, occupation and educational status in both settings.

**Table 4:** Factors contributing for modern contraceptive decision making power in rural areas, Dawro Zone, SNNPR, 2010

**Variables**	**Able to decide on modern contraceptive use**		**AOR (95.0% C.I)**
		
	**Yes**	**No**	
**Knowledge on modern contraceptive**			
**Less knowledgeable**	34(16.3)	177(83.7)	1
**Knowledgeable**	72(58.8)	51(41.2)	6.8 (3.28,13.91)
**Partner's Reaction**			
**Supports my decision**	114(40.0	171(60.0)	1
**Opposes my decision**	7(63.6)	4(36.4)	5.4 (1.20,24.47)
**Don't mind**	3(75.0)	1(25.0)	3.0 (0.27,33.35)
**Don't know**	20(66.7)	10(33.3)	11.1(4.18,29.57)
**Decisions Related To Children**			
**Low involvement**	62(25.6)	180(74.4)	1
**Better involvement**	82(89.1)	10(10.9)	23.3(9.52,56.91)

## Discussion

Knowledge about modern contraceptive method is found to be high in both settings with 99.4% urban and 98.8% rural respondents have heard and can mention at least one method. This figure exceeds the country average reported on Ethiopian demographic and health survey 2005 [3]. This may be attributed to the rural health extension program in the area. Majority of rural women and men in the qualitative study reported that they are not comfortable about long term contraceptive methods. This might be low knowledge about long term modern contraceptive methods. Similar to national report, Condom is frequently mentioned male type in both settings [3]. Current modern contraceptive practice was comparably higher than the national average for Ethiopia [5]. In 2005, a community based research in similar area was reported that contraceptive use was 35% [Bezabih T: Assessment of the determinants of modern contraceptive use in a Dawro community, Ethiopia: submitted]. Relatively higher practice and narrow urban rural difference might be due to the introduction of health extension program and Community Health Agents in all rural kebles of the study area.

As it is true in most African countries, [3,5,14] more than 91% in rural and 77.4% in urban areas were currently using Injectables. This might be due to the availability or acceptability of this method by the community. In this study, majority of rural residents had little knowledge about other types of contraceptive methods; especially long term and permanent methods hence and less utilization of other methods. Even though there are male contraceptive methods options like condom and vasectomy, there was no reported husbands' contraceptive use in both urban and rural settings. Despite 92.4% of women in urban and 91% in rural reported joint decision on contraceptive use, they waited the final say from their husband to use contraceptive. It was also supported by the qualitative finding.

In this study, gender equitable attitude of married women greatly varied between the urban and the rural respondents. Similar findings were found from other studies [11,15]. The fact is usually explained by low educational attainment, economic status with deep rooted cultural belief in the rural areas than the urban women [Bezabih T: Assessment of the determinants of modern contraceptive use in a Dawro community, Ethiopia: submitted, [16,17]].

Ethiopian Demographic and Health Survey of 2000 reported that the independent decision making on the use of modern contraceptive was higher in rural areas than urban area [5]. But this particular study contradicted the EDHS 2000 finding. The finding is in line with low level of communication between partners in rural than urban women. Over half of the respondents have power to decide on modern contraception use with significant difference in urban and rural areas. This implies that, married women who reside in urban area were privileged to decide on the use of modern contraceptive method two and a quarter times more likely than their rural counterparts. This can be explained with the more egalitarian society in urban and patriarchy in rural. Majority of the decisions in such kind of society including family planning is taken by husband due to women's economic dependence, low educational level and existing culture. Similar finding has been reported from Honduras where urban women were more likely to decide on modern contraceptive use than rural women [15].

Modern family planning use decision making did not have significant statistical different with age groups, occupation and educational status. The finding from Honduras depicted as women get older and have more children, male centered decision shown reduction [15] which was also not supported by similar study in Philippines [16]. Decisions related to children and knowledge appears as common predictors for better decision making power on modern contraceptive use in both settings. Having better knowledge about modern contraceptive methods, gender equitable attitude, better involvement in decisions related to children, socio-cultural and family relations are important and significant predictors for better decision making power of women on the use of modern contraceptive methods in urban areas. Whereas in rural areas, knowledge about over all aspects of modern contraceptives, fear of partner's opposition or negligence, involvement in decisions about child were significant contributing factors for better decision making power of women on modern contraceptive use. There was an unusual finding on the perception of partners' reaction on modern family planning which may be attributed to the proportion of women in the categories. In this study, quantitative data was collected only from the women perspective. The validity of the study could have been increased if it has included quantitative data from the male perspective and service providers.

## Conclusions

Current practice of modern contraceptive method was higher than the national and regional figures and the urban rural difference was also reduced in comparison to the regional and national data. In urban setting, gender equitable attitude had significant statistical association with decision making on modern contraceptive use but not in rural settings. Modern contraceptive decision making power found to be higher in urban than rural areas. Increasing knowledge of women on contraceptive will increase contraceptive use decision making power both in the urban and rural areas. Family planning interventions in the area needs to be promoted and consider empowering of women on modern contraceptive use decision making. The role of male partners on women modern contraceptive use decision making needs to be studied systematically from both partners perspective.

## Competing interests

The authors declare that they have no competing interests.

## Authors' contributions

BB, MW and TT designed the study, analyzed the data and drafted the manuscript. EG was involved in the analysis of the data and critically reviewed the article.

All authors read and approved the final manuscript.

## Pre-publication history

The pre-publication history for this paper can be accessed here:

http://www.biomedcentral.com/1471-2458/11/342/prepub
